# Methodological challenges and insights for researching embodied learning in museums

**DOI:** 10.1177/26349795241265258

**Published:** 2024-07-24

**Authors:** Sara Price, Andrew Manches, Robb Lindgren, H Chad Lane

**Affiliations:** 4919 University College London, UK; University of Edinburgh, UK; 14589 University of Illinois Urbana-Champaign, USA

**Keywords:** Body, embodied modes, informal learning, interaction, movement, museum, qualitative

## Abstract

Embodied learning is a nascent term drawing on theories emphasising the role of the body, and body-based interaction in knowledge creation. Whilst embodied learning research has articulated pedagogical and design implications which resonate with museum practice and emphasis on hands-on approaches, translation between embodied learning research and everyday practice is limited. A key challenge is conducting research that is both methodologically rigorous whilst providing tractable implications for complex practice contexts. Whilst this tension is endemic in educational research, the field of embodied learning presents unique challenges. Here, we draw on experiences from a four-year, multisite, academic-practitioner research project investigating embodied learning with young children (3–6 years) in science centres/museums to synthesise, illustrate, and critically reflect on four key challenges: theoretical framing (how embodied learning is conceptualised), nature of the experience (what makes it embodied), evaluating embodied learning, and logistical challenges (capturing multiple modes of interaction, social context, communication). These challenges are illustrated through case studies, contributing a methodological lens for both academics and practitioners investigating the role and implications of embodied learning in museums.

## Introduction

Embodied learning broadly speaks to changes in knowledge, where knowledge is shaped and defined by the body and the body’s role in interaction. Whilst all learning might be described as ‘embodied’, the term is more familiar in pedagogical and design-orientated work (e.g., Lindgren and Johnson-Glenberg, 2013; Nathan, 2021) which draws upon implications from embodied cognition – an umbrella term capturing theoretical claims that cognitive processes are inseparably bound to sensory and motor (sensorimotoric) experiences (e.g., Barsalou, 2008; Lakoff and Johnson, 1999; Wilson, 2002). Embodied cognition has implications for different educational approaches including the role of gesture (e.g., Brooks and Goldin-Meadow, 2016), guidance on the use of physical materials (Pouw et al., 2014), or the design of programs that involve whole-body interaction (e.g., Danish et al., 2020; Price et al., 2016). The context of this paper is research investigating how certain forms of body-based (embodied) interaction may underpin the development of scientific conceptual understanding (e.g., how our experience and interaction with falling objects may underpin understanding of gravity).

Embodied learning research has led to increasing calls to examine implications for everyday practice (Macrine and Fugate, 2021); including museum contexts where design and facilitation are often shaped by initiatives to encourage more ‘active’, ‘kinaesthetic’ or ‘hands-on’ learning. These calls emphasise the need to investigate embodied learning within practice environments, ‘in the wild’; however, whilst the challenges of conducting high-quality, methodologically rigorous research in messy contexts is well-recognised, embodied learning work introduces new challenges that researchers must navigate. The contribution of this paper is to synthesise, illustrate, and critically reflect on four key methodological challenges, drawing on experiences from an international 4-years multisite project on embodied learning in informal learning contexts (‘Move2Learn’), as well as prior work of the team (Lindgren and Johnson-Glenberg, 2013; Nygren et al., 2023; Thomas Jha and Price, 2022).

The four challenges are: (1) *framing of embodiment* (how embodied learning is conceptualised), (2) *nature of the experience* (what makes it embodied), (3) *evaluating embodied learning* (what constitutes knowledge/learning, accounting for context – social and physical), and (4) *logistical challenges* (capturing multiple modes of interaction, social context, communication). Through critical reflection of these four challenges, this paper presents guidance for the design, fieldwork, analysis, and interpretation of future embodied learning research in museum settings, particularly projects seeking to bring together diverse expertise to improve children’s learning through design.

## Move2Learn project

The Move2Learn project involved a collaboration between academics and informal science learning practitioners from six museum sites across the UK and USA – bridging theory and practice (The Frost Museum of Science in Miami, FL, Glasgow Science Centre, London Science Museum, The Children’s Museum of Indianapolis, Sciencenter in Ithaca NY, and Learning through Landscapes, UK). It aimed to advance understanding of embodied interaction for early years informal science learning, which would inform pedagogical design and practical implementation for exhibits and educational facilitation.

Move2Learn drew on recent theoretical work in embodiment (e.g., Abrahamson and Lindgren, 2014; Barsalou, 2008; Lakoff and Johnson, 1999) and academic-practitioner partnerships (Sobel and Jipson, 2015) to investigate sensorimotoric ways that young children interact with and communicate in informal science-related experiences to understand how to improve learning designs and practitioner facilitation, as well as how to capture and evidence learning. Over 3 years (2018–2021), more than 300 children aged 3 to 6 years old were recruited through participating museums, either by prior arrangement through existing members and partner nurseries, or on the day with visitors.

## Research sites

In our discussion of the challenges below, we draw primarily from research at three project sites: Glasgow Science Centre (GSC), the London Science Museum (LSM), and Sciencenter, Ithaca (SI).

**Figure 1.**
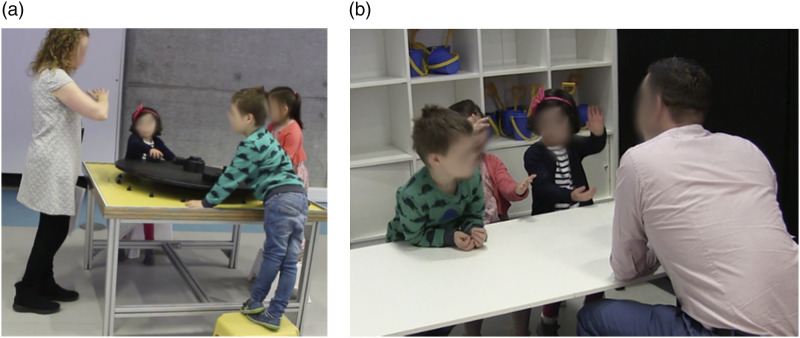
Balance board exhibit and children communicating in interview © authors.

At GSC, we focused on an analogue balance board exhibit (Figure 1), a black wooden circular board (around 1.5 m diameter) which came with equal weight/size blocks. As children tested positioning the blocks, they were exposed to key science mechanisms, including the balancing of forces and the effect of weight and distance from a central pivot. Adults typically assume a supportive role at the exhibit.

**Figure 2.**
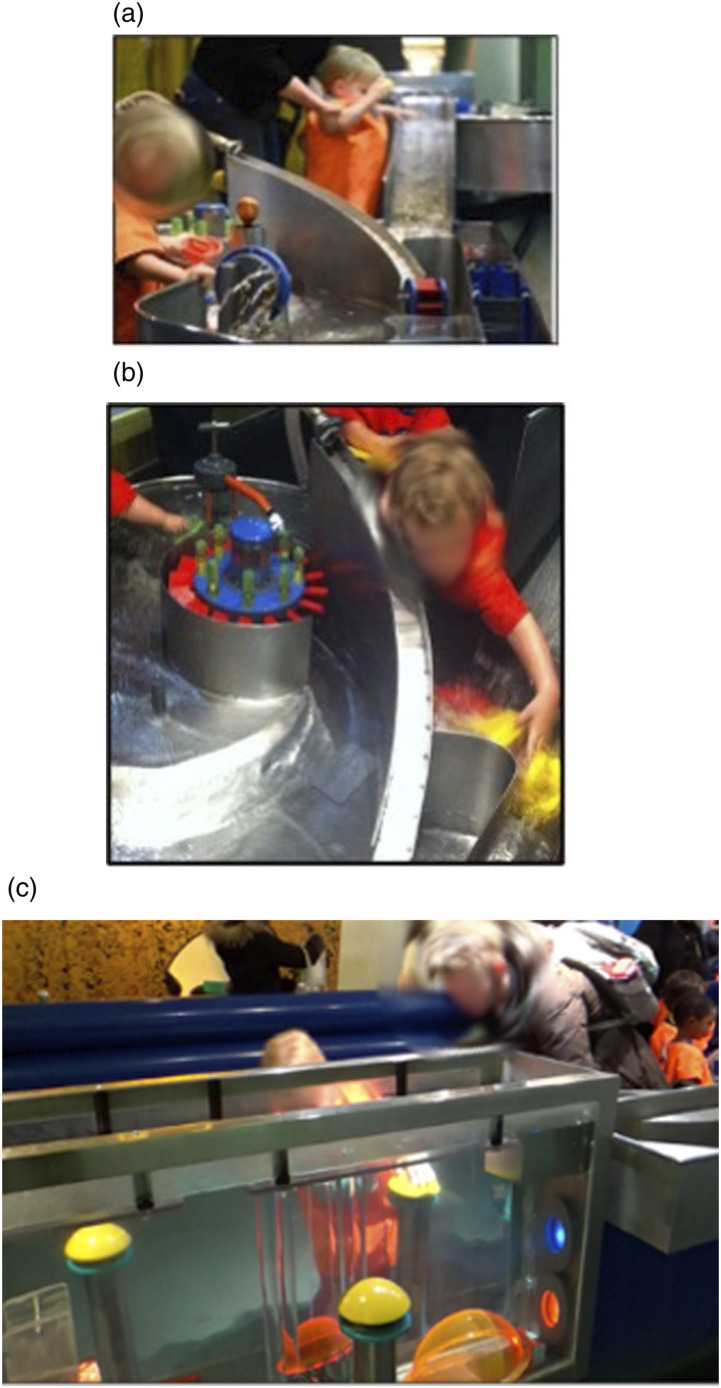
Showing different sections of the water exhibit © copyright authors.

At LSM, we investigated a water-based exhibit comprised of a set of interconnected sections that offered different experiences with water. This included a deep trough to elicit and observe air bubbles rising, a flow of water from higher to lower levels, floating plastic boats, gates to block/limit water flow, water wheels and pumps (Figure 2).

**Figure 3.**
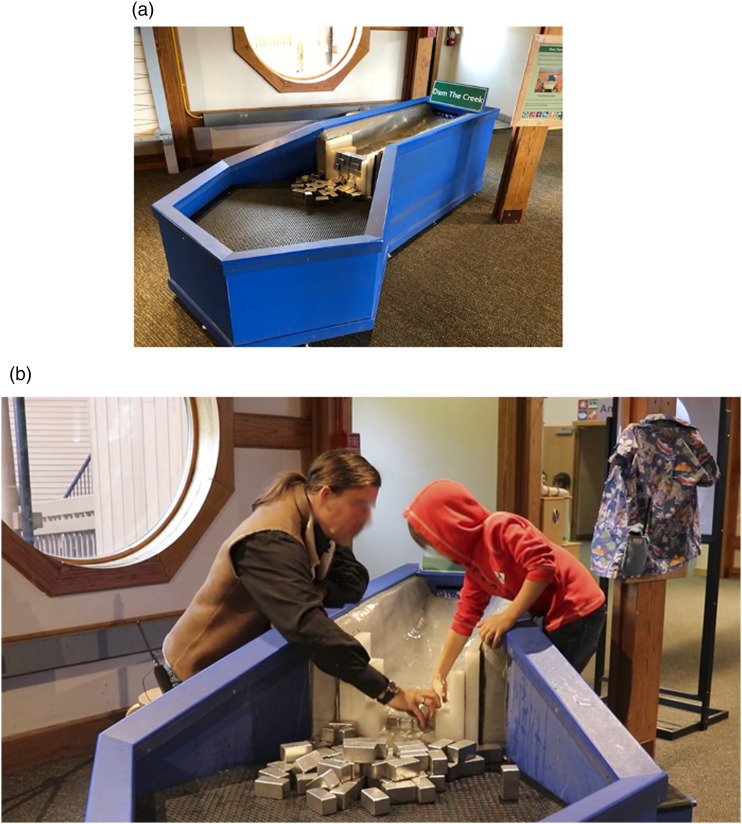
Dam the Creek exhibit and a carer engaging with their child © copyright authors.

At SI, we observed children and their carers engaging with the ‘Dam the Creek’ exhibit (Figure 3), where heavy blocks are arranged to dam the flow of water in a simulated creek. The goal was for children to learn about water pressure and its effects, and how to approach a challenging engineering problem. In all phases of the study, the child’s carer was present.

Many challenges emerged from research within and across sites that are familiar in research-practice partnerships and cross-institutional projects (e.g., site variation, communication strategies, methodological agreement). The focus of this paper is on challenges that were collaboratively agreed as more specific to investigating embodied learning in the wild.

## Challenge 1: Framing of embodied learning

The terms ‘embodied’ and ‘embodiment’ are complex, reflecting how embodied cognition is an umbrella term encapsulating multiple perspectives and claims (see Wilson, 2002). Indeed, an early significant challenge for the project was developing shared meaning for the term ‘embodied learning’. Museum practitioners emphasised the importance of bodily engagement, hands-on interaction, and autonomy in terms of making learning-related choices. Practitioners also emphasised emotional aspects of the learning experiences, such as the importance of physical expressions of engagement or frustration. Researchers on the team adopted a different view that emphasised conceptual development, drawing on research articulating how action experiences can be internalised and subsequently drawn upon across contexts and over time (Nathan, 2021; Barsalou, 2008; Lakoff and Johnson, 1999). These sensorimotoric experiences can often be represented through gesture (hand or whole-body), for example, swinging the forearm with a fixed elbow enacts the motion of a pendulum (Lindgren and Johnson-Glenberg, 2013).

The project team dedicated substantial time discussing different perspectives, ensuring that not one specific viewpoint was privileged. An initial question was whether to avoid the term ‘embodied’ as potential jargon; however, practitioners felt it was important to leverage the term to communicate the novelty of this work given existing comparable initiatives around active learning. With respect to shared definitions, we focused on embodied *cognition*, for various reasons: the term ‘learning’ can sometimes be associated with more formal contexts, and we wanted to allow for perspectives of how embodied ways of thinking can be nurtured to evolve during the project. We consequently defined embodied cognition broadly as a ‘theory claiming that the way we think, communicate, and learn is shaped by our body and our body’s interaction with the world’. This definition allows for the important role of experience, the sensory body, emotion and social interaction for cognition and learning.

In discussing learning in relation to embodied cognition, common ground was found on the importance of understanding specific sensorimotoric experiences. This includes context-specific actions as well as more context-independent gestures (e.g., Alibali and Nathan, 2012). There was similar shared understanding that an embodied learning approach enriches how language is understood and more fully recognises the socially situated nature of meaningful interaction.

To develop a shared sense of embodied cognition in relation to children’s museum interaction, we collectively spent time observing and reflecting on children’s and parents’ exhibit interaction. We identified shared examples relevant to both practitioners and academics to frame our notion of embodied learning and identify ways in which understanding could be revealed through movement. Further, we arrived at the need for an approach to support learning by encouraging sensory and movement experiences that have meaningful connections to the science ideas in the exhibits. These conclusions are aligned with more recently published work on embodied learning (e.g., Nathan, 2021). Here, gesture became a central focus across sites as means to explore how children’s action experiences (e.g., exploring water flow or balance) were subsequently communicated through their hands and bodies. Given the way children and adults, including practitioners, frequently gesture in the museum context, this became a tractable hook for collaborative discussions.

## Challenge 2: Nature of the experience

Ultimately, the goal of exhibit design is to create experiences that elicit particular kinds of interactions that contribute to improved understanding, awareness, or interest. The diversity of designs, and types of interaction they encourage, can challenge an embodied learning approach which more specifically focuses on the way concept-relevant actions map to exhibit features. In some cases, an experience may be highly prescribed, such as inviting a learner to manipulate an object in a specific way to ensure that they will observe the corresponding effect. For example, a wheel or gear with a handle that when turned has visible and meaningful connection to a phenomenon such as a cyclical or mechanical process like the gears in a clock. In other instances, the experience may be more open-ended, to encourage exploration of the problem space. This may involve inviting learners to engage freely with novel physical objects that produce observable effects. The important thing is to be purposeful in the design and ensure that researcher and practitioner perspectives on embodied learning are captured in the result.

At GSC, the balance board exhibit introduced the complex concept of angular relationships between weights and proved to be challenging for young children. Yet, the design provided children with immediate tactile and visual feedback from actively moving blocks. Many children were observed testing their ideas by placing their hands directly on the board to feel the response to different levels of pressure. Across all studies in GSC, children were encouraged to play without prior instruction; however, the collaborative context varied between studies. Children interacted alone or in pairs/dyads, and without an adult or with a parent or facilitator supporting.

In the water exhibit (LSM), children engaged in free exploration without being given any specific goals. The exhibit included several features linked to embodied learning, such as turning the handle of a water wheel, which caused water to be pumped out of a separate outlet, floating plastic boats on moving water, closing and opening gates to dam water or release it, affecting water levels and boats’ movement, and feeling the different states of water flow with hands. Whilst adults were nearby, the number of children present in the confined physical space meant that carers typically observed their children’s interaction with minimal joint engagement.

The dam building exhibit at SI was designed to elicit physical enactments of learners’ ideas about water pressure, flow, and engineering concepts that involve redirection and containment of liquids. The exhibit did not request or require specific actions, which meant the researchers at this site saw more diversity in strategies and corresponding representational gestures for describing those strategies in the post-interview. Feedback was immediate from the exhibit itself, and the water flow was designed to topple any possible dam leading to a ‘dramatic’ conclusion.

## Challenge 3: Evaluating embodied learning

Common across all project sites was the challenge of selecting methods to evaluate embodied experiences. ‘Less work has taken on the task of grappling with how we translate these theoretical insights into concrete methodological tools and approaches.’ (Chadwick, 2017: 55). Research on eliciting embodied experiences through ethnography or sensory methods are often critiqued for their focus on *talk about* the body (e.g., Brown et al., 2011; Chadwick, 2017). In education, methods often focus on traditional disembodied measures that prioritise more symbolic forms of communication, such as language and text. Our work focused differently on understanding the relationship between children’s concrete sensorimotor experiences with science concepts and later reflection about them, where the body was also brought into the reflective process. Our attention was therefore on *process* – how children express meaningful understanding though their interactive experiences and subsequent reflections on those experiences.

Given this, we needed to establish how best to examine children’s conceptual thinking during and following interactive experiences. This posed a challenge, given the age group’s (3–5 years) language level for communicating science ideas. Indeed, language is noted to be inadequate for representing bodily experiences (Brown et al., 2011; Gillies et al., 2004). Previous work shows how gesture is an integral part of meaning making (e.g., Streek, 2009). Children’s sensorimotor experience is shown to shape subsequent gestural communication (e.g., Callinan, 2014; Goldin-Meadow et al., 2001), ‘meaningful’ or congruent action being foregrounded (e.g., Lindgren and Johnson-Glenberg, 2013). Gesture can therefore illustrate children’s understanding (e.g., Sauter et al., 2012) and provide a window into their thinking. Relatedly, the spontaneous use of adult gesture (notably high amongst educators (Perry et al., 1995)), has been shown to support children’s learning both indirectly by helping adults structure their own thinking, and more directly, by presenting action representations that children can internalise to support their own thinking and communication (Alibali and Nathan, 2012).

Given our theoretical stance and focus on how action experiences that foster sensorimotoric representations are used in later reasoning, we drew on this previous work to develop methods for interviewing young children independently (LSM), and jointly with their carers or peers (GSC and SI) that would facilitate bodily and gestural communication about science ideas. Studies in SI conducted pre-as well as post-interviews where researchers asked children about water pressure concepts and solutions they implemented, to look for changes in their ideas that may have come from their embodied interactions. After interaction with the exhibits at all sites, a researcher talked with the children using a semi-structured, child-led interview format, encouraging children to describe and explain their interactive experiences and, where appropriate, their more generalised understanding of the concepts at hand. We aimed to elicit spontaneous representational gesture, alongside talk, in communicating science ideas. To promote spontaneous gesture, we used photo elicitation, noted to be useful in triggering participants’ accounts of embodied and sensory experiences (Orr and Phoenix, 2014). This approach focused children on the exhibit in question and created the opportunity and space for them to reflect and recount their science experiences. We attended to the perceptual and sensorimotor resources which the exhibit invited, how these were taken up by the children and their role in supporting children’s development of science ideas. However, this interview process did not guarantee children would spontaneously gesture; some (a minority) did not. This raises questions over whether to elicit spontaneous gesture or to explicitly ask children to ‘demonstrate’ or ‘show us’ their experience. Yet, ‘demonstrate’, in turn, raises questions over fostering re-enaction of action rather than enabling more abstract gesture or bodily expression to convey embodied knowledge.

Analysis of interviews focused predominantly on spontaneous gestures as a window into how embodied experiences structured children’s thinking. However, differences in analytical approaches emerged between sites, in terms of emphasis and lenses applied to gestural and bodily communication. In GSC, data analysis examined qualitatively and quantitatively the relationship between embodied interaction between child and adult at the exhibit (e.g., number of adult gestures), and subsequently the relationship with gestures children used to communicate their experiences (e.g., the extent to which they abstracted key science mechanisms). Here, we drew upon the educational metaphor of scaffolding to examine and differentiate where adults helped children with specific goals (e.g., eye gaze or pointing to prompt what to manipulate) as opposed to scaffolding ways of thinking more generally (e.g., representational gestures and language to communicate the mechanisms of the board). Whilst post-interaction interviews were typically without adult support, in one study parents were encouraged to support children’s communication ‘as they might naturally’, which revealed insights into adults’ use of embodied communication (e.g., gestures) to help children recollect and describe experiences, including linking to prior embodied experiences (e.g., see-saw).

From LSM, the analytical focus was on the child’s bodily and gestural communication. Whilst accompanying adults were present, they were not explicitly invited to contribute to the interview. Multimodal transcripts (e.g., Jewitt et al., 2016) were produced for each child’s interaction, focusing on how they used their bodies through action, body positioning, movement, tactile exploration, and visual observation to explore science ideas with available objects. Interview transcripts focused on verbal utterances, bodily movement, and gestural forms of communication, e.g., making shapes with their hands, or demonstrating changing speed and direction of movement to convey the meaning they had taken from their experience.

Interviews at SI focused on how children explained their strategies for constructing a dam to address the challenge of water pressure. In post-interviews with children, carers were present but rarely prompted, thus any gestures or statements emerged naturally in interaction with their children. The research team looked for evidence of both an understanding of water pressure (science) and heuristics for addressing the specific challenge of slowing or stopping the flow of water (engineering). Many of the children used gestures to represent the force of water on the structure they were building that did not show up in the pre-interviews. Likewise, the children frequently employed representational gestures to show the strategies – often collaborative strategies that were developed with their carer – that they enacted.

## Challenge 4: Logistical challenges

Whilst academic-practitioner research with young children in the wild generally presents many logistical challenges, these can be accentuated when researching embodied learning, primarily due to demands of capturing nuanced multimodal interaction data across different time points. Here we note the more specific implications for communication/consent, exhibit-research space, data analysis, and academic-practitioner collaboration.

### Communication/consent

**Figure 4.**
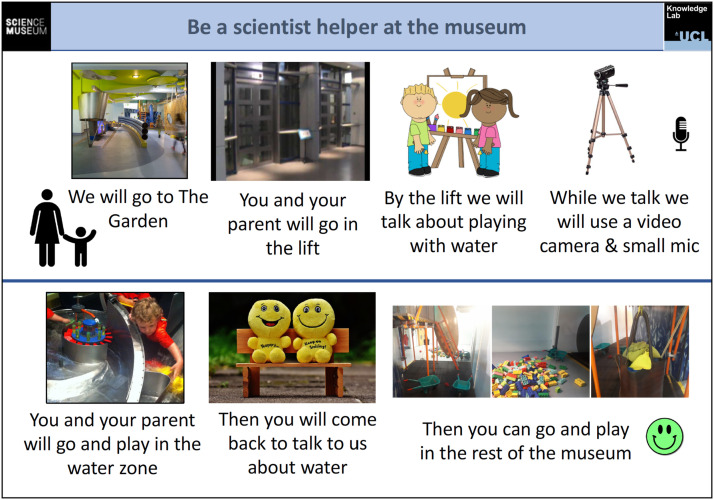
Example consent storyboard used in LSM.

Whilst all research working with young children’s personal data has consent and communication challenges (Dockett et al., 2012), these can be accentuated with the need to video-capture body-based interaction. Here, we developed a picture storyboard of the research process that the researcher used to explain to the child together with their carer (Figure 4). In our studies, caregivers (e.g., parents/teachers) provided written consent to participate and children provided verbal assent alongside their caregivers. The research project was approved by relevant institutional ethics boards. Parents gave signed consent for use of images/video in publications and presentations. For both children and adults, it was important to communicate the focus and goals of the research, whilst minimising the Hawthorne effect – where interaction is changed through awareness of being observed. Consequently, more specific foci, such as body positioning or gesture, were communicated after interaction, with attention to communicating meaningfully to both adults and children.

### Exhibit-research space

The detailed focus on multimodal interaction required the close positioning of one, sometimes two video cameras and the use of attached (wireless) microphones to capture children’s, often quiet, conversations. It was challenging to set up these recording devices safely and minimising their impact on naturalistic interaction. For example, the use of these devices, along with the need for consent, constrained the collection of data on how children’s interaction was influenced by the arrival of, and interaction with, other visitors. The challenge of space was furthered by the need to video record interviews with children after interaction. In busy exhibit spaces this was challenging in terms of managing noise, whilst also minimising the disturbance of participants’ gallery visit (LSM). Separate (quieter) spaces used for interviews away from the exhibit (GSC and SI), facilitated more focused conversations with children, and a chance to reflect on their experiences near, but away from, the exhibit. However, it is important to recognise the challenges of separate spaces, from the time to walk children from the exhibit to interviews, to the need for additional recording equipment. It was also unclear how the change in context influenced children’s recollected experiences.

### Data analysis

As with other multimodal and embodied qualitative research, the analysis of video data is research intensive, requiring close attention to different modes of interaction and communication (Jewitt, 2012). As well as the expertise to integrate and synchronise multiple media (video and audio), it was important to choose analysis software that facilitated collaboration across partners, where we opted for open-source video editing tools (ELAN) to remove financial barriers to practitioner organisations. However, beyond the costs of software is the intense time and training demands of analysing rich multimodal data. For this reason, video sampling was essential and critical in enabling collaborative engagement with practitioners given limited time availability. A deductive sampling approach was taken by academic researchers drawing on the theoretical basis of the research (Derry et al., 2010) and used for shared viewing/analysis where possible. Video sampling therefore focused on episodes of representational gesture or body communication, and related sensorimotor activity in the exhibit interaction.

### Academic-practitioner collaboration

The project presented some specific challenges for the collaboration between academics and practitioners. For practitioners, visitor experience is a priority. This created challenges when fieldwork practices, such as the need for consent forms or the relatively intrusive use of recording equipment, potentially impacted visitor experiences. One key issue was the need to section off exhibits from visitors who were not participating. This issue of visitor experience was arguably accentuated when entry was paid.

Another collaborative challenge reflected the perceived value of methods for capturing and analysing data. The time and resource intensive nature of video analysis typically makes this approach challenging in practice settings where personnel time for such activities is limited, and timely results from research are required. In addition, the lack of clarity about the theory in the field of embodied learning, and its implications for practice, coupled with challenging methods meant the work was sometimes viewed as having academic rather than practical value, challenging the sense of collaborative effort.

## Synthesis and methodological guide

Embodied learning is an exciting and burgeoning approach to pedagogy. Children’s interaction and their embodied experiences are shaped by the design of exhibits or learning activities (particularly those that detect and respond to movement) and/or adult facilitation. The design of these experiences is therefore important. Theoretically grounded research into embodied learning is a powerful tool in informing the design process; yet research in this context presents unique challenges. This article contributes to the field by drawing on a large-scale multisite project to present and explore four key challenges faced in carrying out such research in museums.

**Figure 5.**
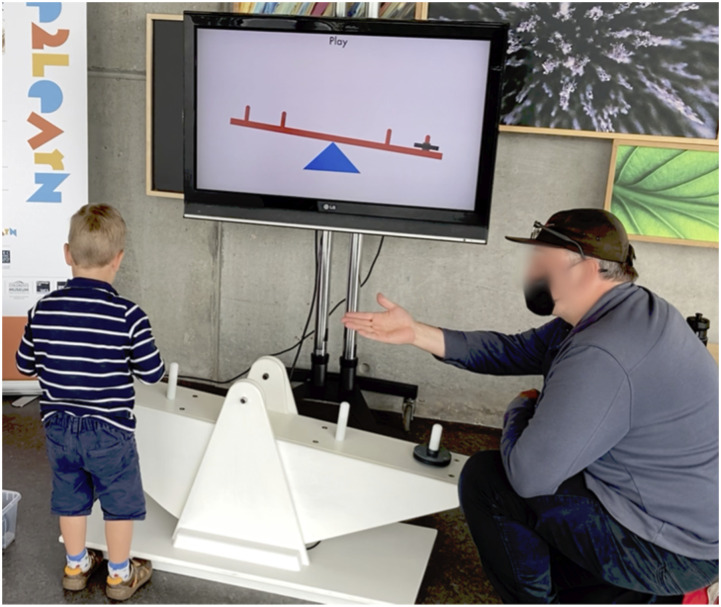
Physical-digital balance beam exhibit © copyright authors.

For the Move2Learn project, theoretical framing and conceptual alignment (Challenge 1) were critical to establishing a foundation for the research both across diverse sites and across academia and museum practitioners. Through many regular group discussions and debates, our definition of embodied learning and development of shared vocabulary was central to ensuring relevance of the research to practice as well as academia, and to provide consistent and coherent underpinning of the different research contexts (e.g., exhibit designs, family units, facilitation opportunities). Our conceptualisation foregrounded the notion of ‘meaningful’ movement in relation to a science idea, thus distinguishing it from more generalised notions of ‘any movement is beneficial’, or notions of ‘kinaesthetic learning’. This conceptualisation was foundational for informing what aspects of an experience make it embodied (Challenge 2), and how to evaluate embodied learning within this framing (Challenge 3). Our research spoke to this by identifying relevant characteristics of the experience and identifying movement that was *meaningful* in underpinning children’s later communication. Our findings revealed the role of nuanced sensorimotoric interaction including actions, body positioning, eye gaze and gestural communication during interaction (example transcripts in Thomas et al., 2021) and extends previous work showing the value of gesture in communication (e.g., Goldin-Meadow et al., 2001) to preschoolers (aged 3–6 years) where gesture conveyed more complexity and level of detail than verbal utterances, providing insight into children’s understanding and focus of attention (Thomas et al., 2021). Interviews revealed interesting differences, from first person simulation of previous actions (e.g., re-enacting moving blocks on a board) to more abstract representations of experience (e.g., using two hands to show balance around a central pivot). Identifying such embodied metaphors offers design implications (Bakker et al., 2012) for practice in terms of encouraging these action experiences, dynamically linking these actions to other information, or encouraging children to reflect upon and re-interpret experiences. This can be achieved through the design of the exhibit itself, or through adult facilitation (e.g., more purposeful use of gestures). Indeed, we explored these design implications in the project through the development and evaluation of a new physical-digital balance prototype exhibit (Figure 5) that aimed to draw attention to and encourage particular actions congruent with the learning goals (see Abrahamson and Lindgren, 2014). Logistical challenges were negotiated through discussions of the team’s values around visitor experience, accessibility, data collection, and data analysis, the kinds of interactions to be observed and spaces needed for such observations, and developing a shared language around issues of consent.

Here we propose a thematic summary (Table 1) from our multisite project to guide future academic-practitioner research work in embodied learning in science museums/centres. The framing of embodied learning provides the foundation of the research direction, shaping how the nature of experiences are identified as embodied, how embodied learning is evaluated, and influencing the logistical challenges of bringing an academic lens into the research space of practice-based contexts.

**Table 1.** Methodological framework for collaborative academic-practitioner embodied learning research in museums.

Embodied learning research in museums				
Research challenges	Proposed steps for addressing challenges			
**Framing of embodied learning**	Discuss what project members value about learning activities/exhibits that involve body movement. What does it mean to learn in these contexts? (e.g., conceptual gains? Emotional development?)	Articulate the available theories/conjectures about the relationship between body movement and learning that are aligned with project goals	Come together to observe children and carers engaged in the kinds of movement the team hopes to elicit; discuss ways that selected theories do and do not apply to these events	Develop a shared vocabulary for describing when and why body movement leads to learning on this project, i.e., what does embodied learning mean for this team?
**Nature of the experience**	Building from the team framing of embodied learning, identify specific actions that have visible and meaningful connections with the target science ideas	Define a set of design features (explicit prompts, object affordances, etc.) that could elicit these actions	Identify opportunities for facilitation: How can carers and/or museum staff be positioned to support these connections	Identify opportunities for reflection: How can children perceive and construct meaning from these connections
**Evaluating embodied learning**	Building from the rationale for the experience design, identify the movements and actions that would suggest progress toward understanding	Building from the rationale for experience design, identify post-experience reflection behaviours (e.g., gestures, verbalizations) that suggest progress toward understanding	Define a set of measurable long-term behaviours/dispositions that could be impacted by students engaging in the experience	Discuss what ‘evidence’ means to the team and what constitutes ‘value’ of embodied designs for learning
**Logistical challenges**	A priori discussions of the team’s values pertaining to visitor experience, accessibility, data collectionetc.	A priori discussion of kinds of interactions the team needs to observe and foreground the kinds of spaces needed to make such observations	Survey available data collection and data management tools in advance; forecast coding approach and calibrate with the volume of data collected	Refine the language the team use around issues of consent; how will the importance of observing body actions be conveyed to children, carers, ethical review boardsetc.

Categorising different methodological challenges is inherently complex given their interconnected nature and difficulty in minimising confounding variables with in situ studies such as those in museums. Different theoretical interpretations will indicate different intervention approaches (e.g., what resources to provide) and the design of studies (e.g., if and how to capture transfer of learning). These in turn will dictate the logistical challenges arising (e.g., recording requirements). Embodied learning research requires recognition of complex interactive contexts, and expertise of practitioners, which may simultaneously sit in tension with the theoretical complexity, need for detailed analysis over themes, and general accountability differences (e.g., visitor experience over publication about learning). Embodied learning, like multimodality, demands recognition of all modes of interaction and communication in meaning making. It recognises that to understand learning requires moving beyond a focus on language and recognising the socially situated nature of meaningful interaction (undertaking ‘in the wild’ studies). For this researcher-practitioner partnership, body action, representational gesture and social engagement were foregrounded to examine different sensorimotor experiences and subsequent communication through gesture (accompanied by speech) to understand the embodied learning process. Methodological processes and challenges within and between sites helped surface generalised themes that inform both research and practice in this growing field.

Whilst the project and this paper focus on informal learning contexts, many challenges are pertinent to more formal contexts (e.g., schools), which likely present other unique challenges (e.g., space for children to express thinking through their bodies or how curricula shapes how knowledge is valued). Critically reflecting on these challenges, and strategies to address, can inform a more comprehensive approach to researching embodied learning in the wild, with the overarching goal of improving children’s learning experiences across contexts.
